# A real-time, gamified approach for optimizing intra-hospital transport of time-critical biospecimens

**DOI:** 10.3389/fdgth.2026.1920674

**Published:** 2026-10-05

**Authors:** Adrian Bundschuh, Annalena Artinger, Rebecca Schönmehl, Christoph Brosius, Jens Langejürgen, Lina Winter, Christoph Brochhausen

**Affiliations:** 1 Medical Faculty Mannheim, Institute of Pathology, University Medical Centre Mannheim, Heidelberg University, Mannheim, Germany; 2 DKFZ-Hector Cancer Institute at the University Medical Center Mannheim, Mannheim, Germany; 3 Circumradius GmbH, Stadthagen, Germany; 4 Fraunhofer Institute for Manufacturing Engineering and Automation IPA, Mannheim, Germany; 5 Division of Rheumatology, Department of Medicine III, Medical University of Vienna, Vienna, Austria

**Keywords:** biospecimen transport, gamification, hospital logistics, pathology, real-time tracking, workflow optimization

## Abstract

Intra-hospital transport of biological specimens is a time-critical, preanalytical step. However, especially for diagnostic or scientific critical biospecimens it remains largely manual, poorly tracked, and decoupled from performance feedback. Existing solutions commonly address isolated parts of the logistics chain but fall short in engaging the personnel who perform it. Therefore, we propose a mobile-first, gamified concept for real-time specimen tracking that combines QR-based documentation with behavioral incentives. Each transport is recorded automatically through start and end scans, closing a gap in the pre-analytical record without adding documentation effort. Beyond external rewards, the concept emphasizes determinants of intrinsic motivation such as task significance to support sustainable engagement. By combining technological and behavioral optimization, this perspective outlines a strategy for time saving, transparent and efficient specimen logistics.

## 1 Introduction

Hospital logistics involves a wide range of processes, including the transportation, storage, and distribution of materials, patients, and biological samples (1). A key component within this field is intra-hospital transportation, which plays an important role in healthcare operations and involves the coordination of multiple resources and processes (2). Effective execution requires precise coordination of personnel, infrastructure, and timing (3).

For transportation of native biological samples, especially for cells and tissue, a fast and efficient workflow is essential. Prolonged cold ischemia time, referring to the period between tissue collection and laboratory processing, is an important pre-analytical factor that may compromise tissue integrity and alter molecular characteristics (4). Consequently, delayed processing can negatively affect the reliability of downstream diagnostic analyses and molecular profiling (5). Minimizing the interval between specimen resection and processing is therefore essential for preserving sample quality, as molecular integrity degrades rapidly under ischemia (6).

In addition, standardized transport and temporary storage procedures are important for maintaining biospecimen quality throughout the pre-analytical phase, particularly when immediate processing is not feasible (7). Moreover, transport delays contribute to prolonged laboratory turnaround times, an important indicator of laboratory performance and service quality (8). Delayed availability of diagnostic results may subsequently affect clinical workflows and potentially delay clinical decision-making. Furthermore, inefficient transport processes may increase waiting times, reduce operational efficiency, and place additional workload on healthcare personnel involved in logistics activities (9).

Efficient transportation processes depend not only on technical infrastructure but also on human factors, including staff motivation and adherence to transport protocols. Transport activities in healthcare environments require the continuous coordination of personnel, resources, and time-critical tasks, making their efficient execution operationally challenging (9). Under such conditions, maintaining motivation and consistent performance can be challenging, particularly when work is repetitive and feedback on performance is limited (10).

Therefore, behavioral incentives offer one approach to encourage more timely and consistent transport. Typical gamification mechanisms include points, badges, leaderboards, achievement systems, or virtual rewards that provide immediate feedback on task completion and performance. Importantly, modern gamification approaches emphasize that sustainable engagement depends not only on external rewards but on supporting intrinsic motivation among the personnel (11). In human resource management, gamification is regarded as a tool to enhance employee motivation and performance (10). However, its application focuses largely on patients, for example in chronic disease management, rehabilitation, and health education (12). Consequently, the use of gamification to support healthcare employees and operational workflows remains underexplored, despite its potential to improve motivation and process performance (13).

The present perspective proposes a new mobile-first, gamified concept for intra-hospital specimen transport that combines real-time tracking with behavioral incentives.

## 2 Current digital solutions for intra-hospital logistics and their limitations

Intra-hospital logistics represents a promising target for digitization. Compared to other industries, the healthcare sector shows a lower level of supply-chain digitalization and the systems currently deployed are commonly fragmented and insufficiently interoperable (2). In this context, a Korean survey on automation investment in healthcare logistics reached only 14.9%, which is less than the cross-industry average (14).

The available systems for intra-hospital logistics can be grouped by their specific function: information systems that manage data and physical methods that transport the samples. Information systems include transport management systems (TMS), laboratory information systems (LIMS), and integrated hospital logistic platforms. TMS are designed to optimize the movement of patients (3). LIMS focus on managing laboratory workflows, including sample registration, intra-laboratory tracking, analysis, and documentation (15). However, these systems do not provide functionality for managing or monitoring sample transport between departments. Integrated hospital logistics platforms aim to coordinate hospital-wide operations such as material flows, inventory, and equipment management within a single system. However, these platforms are complex and resource-intensive to implement, which limits their usability especially in smaller institutions (16).

For the physical transport, pneumatic tube systems are fast and widely used for intra-hospital specimen transportation, particularly for small specimens such as blood samples. This tube system therefore represents an established alternative to manual transport for certain time-critical specimens. However, they are limited to small containers and are therefore unsuitable for larger specimens, including many surgical tissue samples. In addition, concerns have been raised regarding the impact of pneumatic tube transport on specimen quality, particularly for sensitive sample types, although the available evidence remains inconclusive (17, 18). Therefore, pneumatic tube systems are not suitable for larger time-critical tissue specimens, which are the primary focus of this perspective.

Autonomous mobile robots have been established for specimen transport, including time-critical samples from intensive care to the laboratory (19) or pharmacy-to-ward and ward-to-laboratory routes (20). Interestingly, robotic fleets are more efficient in speed and accuracy compared to manual transport (20). However, the system reported by Li et al. required a substantial initial investment (3.5 million RMB = 450,000 €) for ten robots and the supporting infrastructure, alongside structural prerequisites such as wide corridors, elevator integration, and compatible IT systems (20). Given these investment and infrastructure demands, routine deployment of such systems remains limited (2) and most hospitals still depend on manual transport. In pathology, however, specimen dimensions vary considerably and frequently exceed the limitations of pneumatic tube systems. Since robotic transport infrastructure is also rarely available, specimen transport remains predominantly manual. However, relying on human couriers introduces operational challenges that result from the demanding nature of the job itself. Hospital transport personnel face strict schedules and constant pressure of time-critical deliveries, which leads to walking distances from up to 18 miles per day (3).

In general, an end-to-end tracking of samples is not available in the current hospital logistics. In this context, LIMS record specimen registration and automated transport solutions, such as robots, log movement along their routes. However, these data remain isolated rather than connected into a continuous record. Manual transport is typically not captured digitally at all. As a result, the pre-analytical interval from specimen collection to laboratory arrival is rarely measured reliably. This limited traceability contrasts with recommendations from biospecimen research, where exact point-to-point documentation throughout the pre-analytical process has been identified as essential for maintaining sample quality and ensuring reliable downstream analyses (21).

Furthermore, existing systems are oriented toward workflows and hardware, whereas the motivation of the personnel performing manual transport remains unaddressed. Transport performance is therefore decoupled from any form of feedback or reinforcement, despite evidence that feedback, motivation, and engagement can influence employee effectiveness and work performance (10, 22).

Taking these points together, current hospital systems do not adequately support the real-time coordination and transparency of intra-hospital sample transport.

## 3 Proposed system and workflow architecture

To address the identified limitations in current hospital logistics systems, this perspective proposes a specialized, mobile-first application for the real-time coordination and optimization of intra-hospital sample transport. Rather than replacing existing hospital information systems, the proposed solution is designed as a complementary tracking layer that connects TMS, LIMS, and the practical execution of specimen transport. In this way, it supports interoperability across existing hospital systems while enabling the digital documentation of transport events.

The core functionality of the proposed system is based on a lightweight workflow in which each transport is initiated and completed using QR code scans. Start and end timestamps are thereby recorded automatically, enabling precise measurement of transportation duration while simultaneously documenting the pre-analytical transport interval. This eliminates the need for separate manual documentation. From a system perspective, the solution consists of a mobile interface for transport personnel and a backend component for real-time data processing and analysis.

Figure 1 illustrates the transition from the current, largely unmonitored workflow to the proposed QR-based tracking workflow.

**Figure 1:**
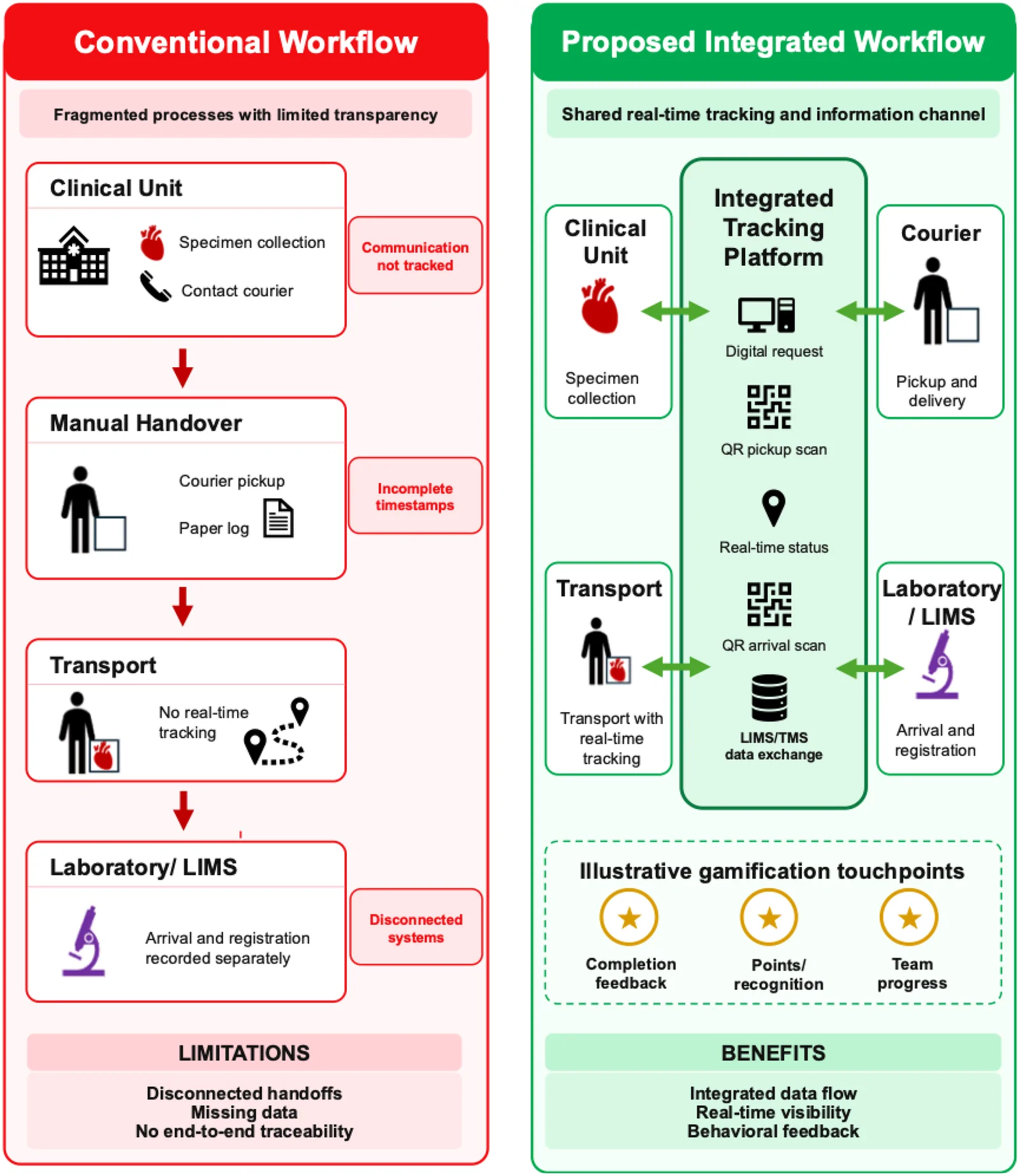
Conventional and proposed workflow. The conventional workflow is a manual process with limited transparency and no real-time tracking. The communication and specimen pickup logging occur manually, making this process prone to errors and increasing the likelihood of missing data. In contrast, the proposed workflow connects the relevant process steps and increases transparency by digitally recording critical events via mobile device. Upon successful specimen delivery, the courier is awarded reward points for potential personal benefits as part of the gamification approach.

## 4 Gamification and behavioral design

Building on the QR-based tracking architecture, the proposed solution includes gamification elements intended to enhance user engagement and promote timely and reliable transport behavior. Transport tasks are associated with reward mechanisms, such as points or virtual incentives, providing immediate feedback on task completion and performance, thereby making individual contributions visible and potentially strengthening perceived competence.

Accumulated rewards can be redeemed for tangible or intangible benefits, allowing users to select incentives that align with their individual preferences and motivations. Providing users with a choice between different incentives may further support perceived autonomy. Thus, rather than treating gamification as a uniform intervention, the proposed design combines different elements intended to address distinct motivational mechanisms, including feedback and competence, individual choice and autonomy, and, through the team-based mechanisms described below, social relatedness (11, 23).

Research on gamified digital tools in healthcare suggests that gamification may positively influence user engagement and motivation, support task completion, and contribute to improved workflow-related outcomes. However, because gamification has generally been embedded within broader digital interventions, its specific contribution remains difficult to isolate (24, 25).

In addition to individual feedback and personal rewards, the concept includes team-based mechanisms through which transport personnel can pursue shared objectives and incentives. Individual transport activities can contribute to a shared team performance, allowing team members to collectively work toward predefined goals and associated rewards. Rather than focusing exclusively on individual performance, this approach is intended to encourage cooperation and collective responsibility for timely specimen transport. Shared achievements may provide social recognition and strengthen perceived relatedness among team members, thereby making successful transport performance a collective rather than solely individual objective (23).

Contextual information about the transported specimen represents a complementary behavioral component rather than a gamification mechanism itself. The system may provide transport personnel with information about why a specimen is clinically or scientifically relevant and how timely transport contributes to its diagnostic or research purpose. By making the significance of the transport task more explicit, this information may increase perceived task significance and experienced meaningfulness at work, thereby supporting intrinsic motivation beyond the external incentives provided through gamification (22).

In contrast to conventional task allocation models, where faster task completion may lead to increased workload without direct benefit to the individual, the proposed system introduces explicit reward mechanisms that positively reinforce efficient task execution. By aligning individual performance with tangible incentives, the system aims to mitigate negative perceptions of workload imbalance and foster sustained user engagement. Importantly, the system is designed as a purely incentive-based approach. Its use is voluntary, and no penalties are associated with non-participation. The aim is to provide positive reinforcement without introducing pressure or negatively affecting existing workflows. By reinforcing timely and reliable transport processes, the system supports both user acceptance and specimen quality.

Together, the tracking architecture and behavioral design combine technological and motivational approaches within a single transport workflow. By aligning operational objectives with user motivation through gamification, the proposed system aims to bridge the gap between system-level coordination and the practical execution of time-critical specimen transport.

## 5 Discussion

This Perspective proposes a mobile-first approach that combines real-time, QR-based specimen tracking with behavioral design elements to address gaps in the transparency, coordination, and documentation of time-critical intra-hospital specimen transport. As a conceptual framework without empirical validation, its potential operational and clinical benefits must be considered alongside implementation challenges, behavioral risks, and the need for prospective evaluation.

### 5.1 Expected operational and clinical impact

The successful adoption of digital solutions in healthcare environments depends not only on technical performance but also on user acceptance, organizational support, and clear value propositions for different stakeholder groups (26). The proposed system therefore targets hospitals, pathology departments, and clinical laboratories in which time-critical specimen transport represents an important component of diagnostic workflows. Relevant stakeholders include hospital management, laboratory personnel, and transport staff, who may benefit from increased process transparency and more efficient workflow coordination.

From an operational perspective, improved specimen transport may be particularly relevant for intraoperative frozen-section specimens, which require timely transfer from the operating room to the pathology laboratory. Delays, misdirection, or incomplete handovers may prolong the frozen-section workflow and delay communication of diagnostic findings required for intraoperative decision-making (27). Real-time tracking and improved coordination may reduce avoidable delays and uncertainty while supporting more reliable workflow execution. Task-relevant information, such as optimal routes, opening hours of receiving units, and relevant contact persons, may further reduce coordination effort.

From a clinical perspective, timely and reliable transport may contribute to shorter diagnostic turnaround times while limiting avoidable pre-analytical delays that can affect specimen quality (4–9). From a research perspective, controlled transport conditions may similarly support the quality of biological specimens used for complex downstream analyses. The resulting operational, clinical, and economic effects should be evaluated prospectively in clinical practice.

Beyond technological process optimization, the proposed approach explicitly considers human factors by combining tracking functionality with behavioral mechanisms. This may facilitate user engagement and align individual transport behavior with organizational objectives. The hypothesized relationship between tracking, motivation, transport performance, and clinical outcomes is summarized in Figure 2.

**Figure 2:**
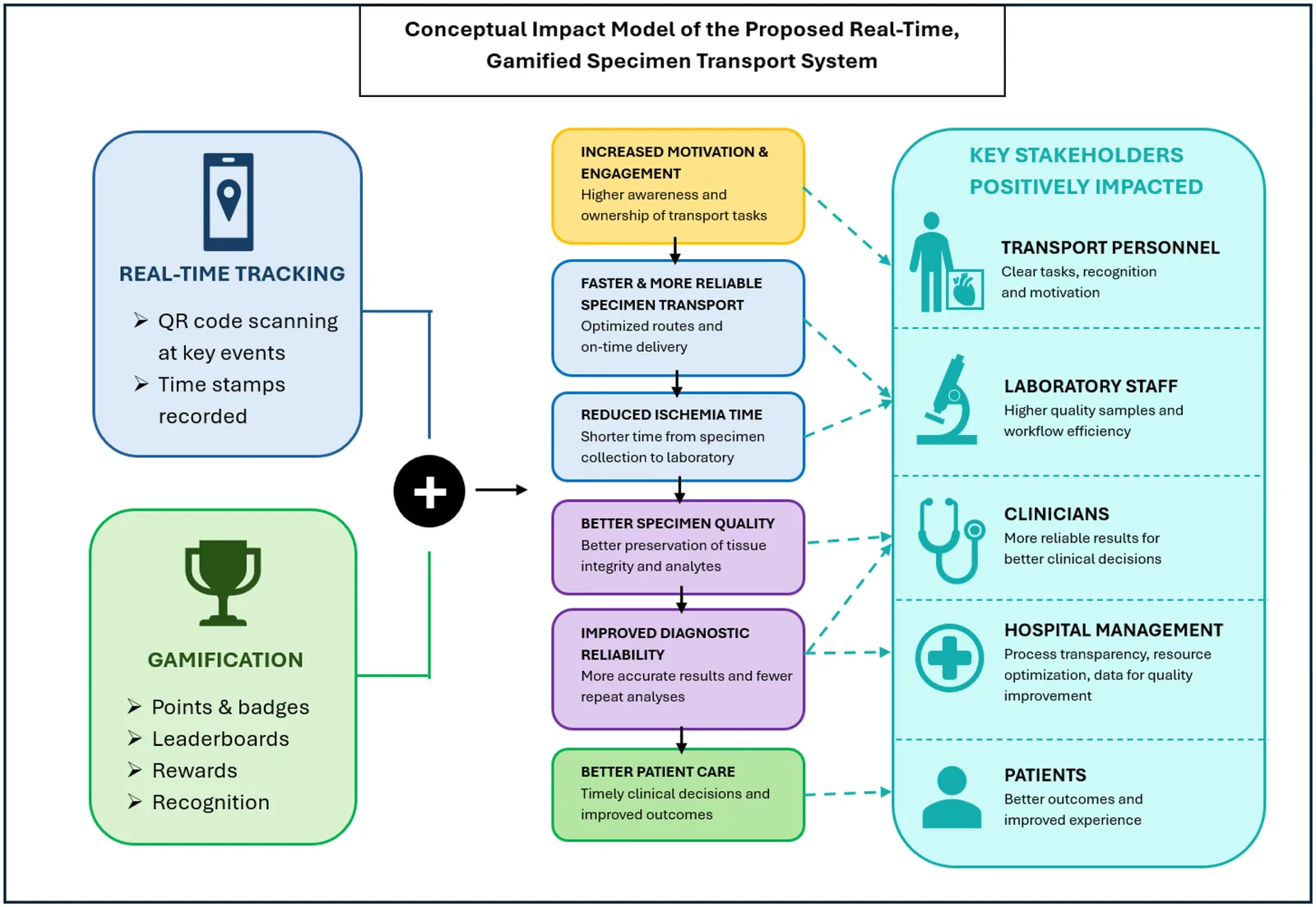
Conceptual impact model of the proposed real-time, gamified specimen transport system. Real-time tracking and gamification are combined to support motivation and engagement, reliable specimen transport, reduced ischemia time, specimen quality, diagnostic reliability, and ultimately patient care. These effects are expected to provide benefits for transport personnel, laboratory staff, clinicians, hospital management, and patients. The gamification components shown represent illustrative examples rather than an exhaustive set of possible game elements.

### 5.2 Limitations, risks, and mitigation

Despite its potential benefits, the proposed approach also introduces challenges that should be carefully considered. Gamification mechanisms need to be designed in a way that avoids excessive competition, stress, or prioritization of speed over proper specimen handling. In time-critical workflows, faster transport must not compromise safety, documentation quality, or compliance with clinical protocols. Therefore, gamified performance indicators, such as transport time or task completion, should not be equated with clinical or operational success, but should be interpreted alongside safety, handling quality, and protocol compliance.

In addition, the accuracy and reliability of collected tracking data depend on correct and consistent user interaction. Incomplete, incorrect, or intentionally manipulated QR code scans could reduce data quality and distort performance evaluation. Incentive-based systems may also lead to unintended behaviors, such as unsafe rushing, strategic selection or avoidance of particular transport tasks, or uneven workload distribution. Reward opportunities may further differ depending on factors outside an individual's control, such as transport routes, task difficulty, or workload. Mitigation should therefore include safeguards against implausible scan patterns, consideration of task-specific conditions when evaluating performance, and reward structures that avoid systematically disadvantaging personnel assigned to more demanding transport tasks.

Integration into existing hospital IT infrastructures may also require attention to interoperability, data protection, and user acceptance. Organizational factors, including staff training, workflow adaptation, and acceptance by clinical personnel, represent additional challenges for real-world implementation. Accordingly, the system should be implemented as a complementary layer that supports existing workflows rather than replacing established clinical or laboratory systems.

Finally, the effectiveness of the proposed approach depends on its successful adoption and long-term use. Quantitative evaluation in real clinical environments is necessary to assess its impact on transport efficiency, specimen quality, and user behavior, and potential unintended effects of the incentive mechanisms.

### 5.3 Evaluation and future research

The proposed concept should currently be regarded as a conceptual framework that requires validation in real-world clinical environments. The present work does not include empirical data on transport performance, user acceptance, or specimen quality outcomes. Future studies will therefore be necessary to determine whether the combination of real-time tracking and behavioral incentives leads to measurable improvements in transport performance, user engagement, and specimen quality.

Future evaluations should address multiple complementary domains. Usability and user experience should be assessed in terms of usability, perceived workload, and barriers to routine use, while implementation outcomes should include acceptability, appropriateness, feasibility, adoption, accuracy, and sustainability. Behavioral and process-related outcomes may include compliance with the proposed workflow, response and completion times, handoff reliability, and task prioritization. Operational and clinical outcomes should include transport and turnaround times, avoidable delays, specimen integrity, rejection rates, and transport-related errors. These outcomes should be evaluated alongside potential economic effects and unintended behavioral consequences of the incentive mechanisms (12, 26).

The proposed concept highlights the potential value of combining technological and behavioral optimization in healthcare logistics. Gamification has demonstrated positive effects on motivation, engagement, and performance in other contexts (10, 28), while its application to healthcare operations remains comparatively limited (13). Future research should therefore investigate how specific behavioral design elements interact with clinical workflows and organizational conditions and whether such approaches can sustainably support healthcare personnel and workflow optimization.

## Data Availability

The original contributions presented in the study are included in the article, further inquiries can be directed to the corresponding author.
